# Can requests for real-world evidence by the French HTA body be planned? An exhaustive retrospective case–control study of medicinal products appraisals from 2016 to 2021

**DOI:** 10.1017/S0266462324000291

**Published:** 2024-05-17

**Authors:** Judith Fernandez, Céleste Babin, Camille Thomassin, Floriane Pelon, Sophie Kelley, Pierre Cochat, Margaret Galbraith, Driss Berdaï, Antoine Pariente, Francesco Salvo, Antoine Vanier

**Affiliations:** 1 HTA Department, Haute Autorité de Santé, La Plaine Saint-Denis, France; 2 Scientific Board and Chairman of the Transparency Committee, Haute Autorité de Santé, La Plaine Saint-Denis, France; 3 CHU de Bordeaux, Pharmacoepidemiology and Appropriate use of Medicine Team, Public Health Department, Clinical Pharmacology Unit Bordeaux, Nouvelle-Aquitaine, France; 4 University of Bordeaux, INSERM, BPH, U1219, Team AHeaD Talence, Aquitaine, France; 5 CHU de Bordeaux, Regional center for pharmacovigilance Public Health Department, Clinical Pharmacology Unit Bordeaux, Nouvelle-Aquitaine, France; 6 Université de Tours, UMR U1246 Sphere, Inserm Tours, Centre-Val de Loire, France

**Keywords:** real-world data, real-world evidence, life-cycle assessment, postlaunch evidence generation, health technology assessment

## Abstract

**Objectives:**

In France, decisions for pricing and reimbursement for medicinal products are based on appraisals performed by the National authority for health (*Haute Autorité de Santé* (HAS)). During the appraisal process, additional real-world evidence can be requested as “Post-Registration Studies” (PRS) when there are uncertainties in evidence that could be resolved by additional data collection. To facilitate PRS planning, a retrospective exploratory analysis was conducted to identify the characteristics of medicinal products associated with a PRS request.

**Methods:**

This analysis encompassed all appraisals finalized between January 1, 2016 and December 31, 2021 and compared products for which the appraisal led to a PRS request with those that did not.

**Results:**

Six hundred positive opinions for reimbursement were identified, with a PRS request present in 17 percent (n = 103) of cases. The independent characteristics associated with a PRS request were a mild or moderate clinical benefit score, a major to moderate or minor clinical added value score, previous availability under an early access program, and certain therapeutic areas (neurology, pulmonology, and endocrinology). These findings suggest two different profiles of PRS requests: (i) products for which there is uncertainty in the size of the clinical benefit and (ii) innovative products for which a substantial benefit is expected but uncertainties persist.

**Conclusions:**

These results will assist health technology developers to better anticipate data generation to promptly address uncertainties identified by HAS. It may also help HAS and other assessment agencies to work together to improve postlaunch evidence generation according to the characteristics of the medicinal products.

## Introduction

Access to reimbursement for medicinal products in France requires pharmaceutical companies to submit a dossier to the National Authority for Health (*Haute Autorité de Santé* (HAS)), which is responsible for Health Technology Assessment (HTA) (1;2). Appraisals performed by HAS impact reimbursement and pricing decisions taken by the Minister of Health, as well as the level of reimbursement by the National Health insurance. They are grounded in the principles of evidence-based medicine and a legislative framework, as per the Rules of Procedure (3;4).

At the time of initial assessment and appraisal, which typically follows the marketing authorization, pivotal clinical trials are the primary source of submitted data. Randomized controlled trials (RCTs) remain the gold standard for establishing efficacy (5); however, RCTs require strict implementation conditions and an optimal management setting that deviate from routine care (6;7). To mitigate potential biases that could compromise internal validity and to accommodate pragmatic and ethical constraints, RCTs are conducted in selected and homogeneous populations that only partially mirror the characteristics of patients treated in routine care. Additionally, the use of surrogate endpoints and constraints on study duration may also limit the applicability of results (8;9). Consequently, HAS often identifies uncertainties regarding a trial’s internal and/or external validity. In cases where major uncertainties need to be resolved, HAS can request pharmaceutical companies to collect additional real-world data in “Post-Registration Studies” (PRS) (10). PRS requests are formulated on a case-by-case basis by HAS in its final appraisal document. The request may cover one or more research questions related to: patients’ and prescribers’ characteristics, conditions of use, safety and/or effectiveness, including patient-reported outcomes. The appraisal document systematically outlines the timeframe for expected PRS results and reassessment of the product and can provide recommendations on the study design or data source to be used. A protocol assistance procedure is proposed by HAS to further help health technology developers in conceptualizing the design of the PRS according to the request (11). Similar postlaunch data collection exists in other HTA jurisdictions (12). Germany, for example, amended the AMNOG law in 2019 to allow the Federal Joint Committee (G-BA) to request nonrandomized comparative studies under certain conditions where the clinical benefit cannot be quantified (13–15). Other mechanisms for postlaunch evidence generation (PLEG) required by HTA agencies are often linked to outcomes-based managed entry agreements (16–20).

Although it has been possible to request PRS in France since the establishment of the appraisal committee, there is limited data available regarding its role in HAS’s assessments and appraisals (21).

The utilization and the sources of real-world data are increasing, exerting an influence on HTA and emphasizing the necessity to further delineate the role of PRS in the French system (22–24). For example, the use of electronic health records enhances the conduct of large-scale observational studies (25;26). In the meantime, the increasing reliance on accelerated clinical development strategies, which hinge on single-arm trials as pivotal studies, diminishes the strength of evidence and consequently amplifies uncertainties regarding a medicinal product’s effectiveness at the time of registration (5;27–29). This conceptual shift has led to profound changes in expectations associated with real-world evidence. Initially, real-world evidence was primarily needed to bridge knowledge gaps associated with robust and well-conducted RCTs (mainly regarding safety or applicability of results as previously mentioned). However, they are now expected to provide robust postlaunch information to complement the limited evidence, both in quantity and certainty of results, provided by manufacturers in the context of accelerated developments. The ability of real-world evidence to adequately inform conclusions on relative efficacy and safety is, however, still a subject of debate (30). Nonetheless, HAS has taken several steps to adapt to these evolving circumstances and enhance the quality of PRS and postlaunch evidence in general. The HAS recently initiated a census to identify relevant data sources that can be used by health technology developers for PRS (31). A methodological guideline on real-world data was published in 2021 to support robust evidence generation for HTA (32). Furthermore, the monitoring of medicinal products in routine care has been incorporated into the HAS action plan for innovative products to assess the fulfillment of their promises (33). The need for routine care monitoring is further reinforced by the emergence of advanced therapies with high potential but high financial cost and methodological uncertainties (34). For such products, it is crucial to promptly close evidence gaps to ensure that patients receive beneficial treatments, and that the national health insurance budget is wisely allocated. Recent appraisals by HAS of CAR-T cells are a good example of PRS requests with close monitoring of the data obtained in clinical practice. In these instances, annual submissions of real-world evidence were requested to monitor the product’s effectiveness in routine care and adjust appraisals (35;36).

Overall, while results from PRS were traditionally provided to HAS years after their request, it has now become imperative to accelerate real-world evidence generation by proactively planning these studies during the clinical development program. To support pharmaceutical companies in this approach, this study was conducted with the aim of identifying the characteristics of medicinal products associated with a PRS request by HAS.

## Methods

### Study design and data source

A retrospective exploratory case–control study was conducted to identify the characteristics of medicinal products for which the appraisal resulted in a PRS request versus those that did not. This analysis encompassed all requests for reimbursement finalized between January 1, 2016 and December 31, 2021. Two types of dossiers were excluded from this analysis as they do not lead to a PRS request: dossiers assessed through a simplified procedure (typically intended for generics or biosimilars) and dossiers that obtained a negative opinion for reimbursement.

Data were extracted from the HAS information system (EVAMED®) that mainly summarized data publicly available in the published appraisals. For this analysis, the query to extracted data was made by one of the authors (JF) and checked by the specialized project manager in charge of EVAMED®. Since the use of this information system is mandatory for all assessment dossiers, it was assumed that the database comprises all dossiers that met the eligibility criteria for the study. Consequently, no missing data were anticipated.

#### Outcome and characteristics to be investigated: definition and selection

The outcome of the study is the occurrence of a PRS request or not. A qualitative assessment of the internal HAS database was performed to identify any relevant characteristics that might be linked to this outcome (e.g., by removing variables with an administrative purpose only; the original full list of variables of the internal database is available as an online supplementary file (eTable 1)). The working hypothesis was that two distinct profiles could potentially be associated with a PRS request: (i) products for which the patient benefit seems questionable and (ii) innovative products for which a substantial benefit is anticipated, but uncertainties persist. This hypothesis was primarily based on preliminary work conducted by HAS (37 *;* 38) and a subjective assessment by some investigators of the study, as the HTA department has a long experience in PRS requests. Overall, 12 characteristics were selected for investigation. Table 1 provides comprehensive definitions and outlines how these characteristics were quantified as variables for the subsequent statistical analyses. It is acknowledged that some characteristics used in this analysis are unknown during research and development phases of a new medicinal product (marked with an footnote a in Table 1). However, guidelines from the regulator and/or HAS can support the health technology developers in anticipating these decisions, scores, or statuses (3 *;* 39–41). Discussion during early dialogues or joint scientific consultations can also help the developer to foresee these characteristics and therefore the request for a PRS.

**Table 1.** Definition and measure of the investigated characteristics of the study

Characteristic	Definition	Measure
Indication type	Dossiers are assessed by indication. This characteristic differentiates a first marketing authorization from an extension of indication.	Dichotomous: first indication *versus* extension of indication
Registration type	There are two registration lists for medicinal products coverage in France: a list restricted to hospital use and a list for outpatient prescription. Depending on the terms or restriction of delivery or use stipulated in the marketing authorization decision, pharmaceutical companies may request registration on one or two list(s).	Dichotomous: hospital use only *versus* both outpatient prescription and hospital use. Registration on the list for outpatient prescription only being exceptional, these cases were classified in the category “outpatient setting and hospital use.”
Therapeutic area	This characteristic classifies dossiers according to the therapeutic area the indication is targeting.	Categorical in five categories: Oncology, Neurology, Endocrinology (including diabetes), Pulmonology, Other. The internal HAS database proposes 32 categories. Only the four with a prevalence of at least 5% of the dossiers (Post–Registration Studies or no Post–Registration Studies request) were retained. Other dossiers were merged in one category (Other).
Advanced therapy medicinal products (ATMP)	A specific framework overseeing medicinal product based on genes, tissues or cells according to the regulation	Dichotomous: yes/no
Orphan designation	Medicinal products targeting rare and serious diseases are framed by the EU orphan regulation	Dichotomous: yes/no
Pediatric indication or formulation	This characteristic informs on whether the indication targets a pediatric population, where evidence available for appraisal is often scarcer or of a lower level of certainty of results than for adults.	Dichotomous: yes/no Indication covering both adults and pediatric are classified as not pediatric.
Conditional marketing authorization/marketing authorization under exceptional circumstances^a^	According to the European Medicines Agency, medicinal products can be licensed based on less comprehensive data than usually required by the regulator if targeting unmet medical need (e.g., through a conditional marketing authorization or a marketing authorization under exceptional circumstances).	Dichotomous: yes/no
Previous national early access program^a^	Medicinal products presumed to be innovative and addressing unmet medical need may be available in France before coverage by common law or even sometimes before the marketing approval though an early access program upon HAS decision.	Dichotomous: yes/no
Clinical benefit (CB)^a^	The CB is an assessment of the intrinsic value of the drug and provides an appraisal for its inclusion on the reimbursement list. According to the French legislation, the CB considers five criteria (4): the severity of the disease; the drug’s clinical efficacy and safety; the therapeutic aim (preventive, symptomatic or curative); the drug’s place in therapeutic strategy and the impact in terms of public health. The CB is graded in four categories: “substantial,” “moderate,” “mild” or “insufficient.” An “insufficient” CB corresponds to an unfavorable opinion for the drug’s inclusion on the reimbursement list. A “substantial,” “moderate,” or “mild” CB implies that HAS has a favorable opinion for registration on the list of reimbursed products and provides a recommendation on its level of co–payment.	Categorical (three categories): Substantial Moderate Mild In case HAS concluded to different levels of CB for multiple indications for the same medicinal product, only the highest level was kept for the statistical analysis. Dossier which obtained an “insufficient” CB in the entire indication were excluded from the analysis as they cannot lead to a post–registration study request.
Public health impact^a^	This characteristic is one of the five criteria of the CB. This criterion was considered relevant for this analysis as it captures the collective impact of medicinal products and may affect the pricing process.	Dichotomous: yes/no
Clinical added value (CAV)^a^	The CAV assesses the clinical improvement achieved by use of the medicinal product compared with existing therapies or best applicable care. It is one of the key elements for pricing negotiation. CAV is graded in five categories: “major,” “important,” “moderate,” “minor,” or “absent.” HAS gives a “major” to “minor” CAV according to the drug superiority in terms of efficacy and/or tolerability as compared to the current therapeutic strategy. An “absent” CAV is given whenever there is no gain observed as compared to the existing therapies.	Categorical (three categories): Major, important, and moderate Minor Absent Major to moderate CAV were grouped in one category as their consequences on pricing are globally similar. In case HAS concluded to different level of CAV for one indication, only the highest score was kept for the analysis.
Exceptional medicinal therapy^a^	Expensive medicinal products used in outpatients setting and/or with a precise indication can be registered in a specific list of “exceptional medicinal products” according to the French regulation.	Dichotomous: yes/no

a These characteristics are not known during research and development phases of a new medicinal product. However, guidelines from the regulator and/or HAS can help the health technology developers to anticipate these decisions, scores, or statuses.

### Statistical analysis

Categorical variables were described as frequencies by counts and percentages. Although the dataset is exhaustive for the period considered in the study, comparisons were performed by means of statistical hypothesis testing as the dataset can be viewed as a sample from a broader population of dossiers from a more extended timeframe. The selection of a final multivariate logistic regression model with PRS request as the dependent outcome was performed through a two-step procedure. First, univariate comparisons of all collected characteristics in relation to the presence or absence of a PRS request were made by fitting univariate logistic regression models. To be sensitive, characteristics with a nominal p-value against the null hypothesis of a beta regression coefficient equal to zero less than 0.25 were included in a multivariate logistic regression model. Characteristics with fewer than 10 observations in at least one group were excluded to avoid estimation issues. Characteristics that were retained after this first step were used to perform the second step which consists in selecting a multivariate logistic regression model through an iterative procedure. Specifically, the selection of the final multivariate model was performed through a stepwise backward procedure with a selection criterion that was a combination of likelihood-ratio test and analysis of the relative change of beta parameters. A characteristic was retained if, after being excluded from the model, either the p-value of the likelihood-ratio test was lower than 0.05 or the relative change associated with at least one of the beta parameters of the other characteristics exceeded 10 percent. Since these analyses were exploratory, no control for multiple hypothesis testing was applied (42). Parameter estimates of the final model were used to compute adjusted odds ratio (ORa) and confidence intervals at a 95 percent level (95 percent CI). Characteristics for which ORa 95 percent CI excluding the value of no effect (i.e., 1) in the final selected model were identified as characteristics independently associated with a PRS request (i.e., corresponding to a nominal alpha level of 5 percent). As a supplementary analysis, a search for effect modification was performed through systematic tests of first-order interaction terms between the characteristics retained in the final multivariate logistic regression model. Details on the method and results of these supplementary analyses are provided in an online supplementary file (eText 1).

Statistical analyses were performed using SAS statistical software version 9.4 (SAS Institute Inc).

## Results

Between January 1, 2016 and December 31, 2021, HAS published 600 positive opinions for reimbursement of a new medicinal product (325 dossiers) or an extension of indication (275 dossiers). The 275 dossiers for an extension of indication concerned 181 proprietary medicinal products. For 132 proprietary medicinal products, HAS issued an appraisal of only one extension of indication. For 49 medicinal products, more than one extension of indication was assessed by HAS during this timeframe (median number of appraisals = 2, inter-quartile range = [2–3], maximum number of appraisals for the same medicinal product = 12). In about 17 percent (n = 103) of these appraisals, a request for PRS was identified.

HAS appraises the clinical value of medicinal products using two scores: the clinical benefit and the clinical added value (refer to Table 1 for definitions and scoring details). Table 2 provides a detailed description of the characteristics of these 600 appraisals obtained from the HAS database. In the majority of cases (81.6 percent, n = 490), the medicinal product obtained a substantial clinical benefit score, and HAS concluded there was no added value (55.5 percent, n = 333) for the considered indication(s). The most frequently encountered therapeutic areas were oncology (27.2 percent, n = 163); neurology (8.0 percent, n = 48); endocrinology (7.0 percent, n = 42); and pulmonology (5.7 percent, n = 34). Since the prevalence of other therapeutic areas was less than 5 percent, they were combined in one category (“other” – 52.2 percent n = 313) to facilitate statistical analyses.

**Table 2.** Global characteristics and univariate comparisons depending on the request of a post-registration study

Characteristic	Total *n (%)*	Files with a PRS request *n (%)*	Files without a PRS request *n (%)*	p-Value
**General characteristics**				
**Indication type**				0.002
*First indication*	325 (54.2)	70 (68.0)	255 (51.3)	
*Extension of indication*	275 (45.8)	33 (32.0)	242 (48.7)	
**Registration type**				0.51
*Hospital use only*	233 (38.8)	37 (35.9)	196 (39.4)	
*Outpatient setting and hospital use*	367 (61.2)	66 (64.1)	301 (60.6)	
**Therapeutic area**				<0.001
*Oncology*	163 (27.2)	19 (18.4)	144 (29.0)	
*Neurology*	48 (8.0)	13 (12.6)	35 (7.0)	
*Endocrinology (including diabetes)*	42 (7.0)	14 (13.6)	28 (5.6)	
*Pulmonology*	34 (5.7)	16 (15.5)	18 (3.6)	
*Other*	313 (52.1)	41 (39.9)	272 (54.8)	
**Regulatory characteristics**				
**Conditional MA or MA under exceptional circumstances:** *Yes*	44 (7.3)	14 (13.6)	30 (6.0)	<0.001
**Orphan designation:** *Yes*	136 (22.7)	38 (36.9)	98 (19.7)	<0.001
**ATMP:** *Yes*	15 (2.5)	10 (9.7)	5 (1.0)	<0.001
**Pediatric indication or galenic:** *Yes*	212 (35.3)	35 (34.0)	177 (35.6)	0.75
**Exceptional medicinal products:** *Yes*	30 (5.0)	8 (7.8)	22 (4.4)	0.16
**Previous early access program:** *Yes*	129 (21.5)	35 (34.0)	94 (18.9)	<0.001
**Appraisal of the medicinal product by HAS**				
**Clinical benefit**				<0.001
*Important*	490 (81.7)	68 (66.0)	422 (84.9)	
*Moderate*	57 (9.5)	15 (14.6)	42 (8.5)	
*Mild*	53 (8.8)	20 (19.4)	33 (6.6)	
**Clinical added value**				0.11
*Major, important, or moderate*	96 (16.0)	22 (21.4)	74 (14.9)	
*Minor*	171 (28.5)	33 (32.0)	138 (27.8)	
*Absent*	333 (55.5)	48 (46.6)	285 (57.3)	
**Public health impact:** *Yes*	118 (19.7)	22 (21.4)	96 (19.3)	0.63

Abbreviations: ATMP, Advanced therapy medicinal product; MA, market authorization; PRS, post-registration study.

Twelve characteristics were tested for an association with a PRS request through univariate analyses (refer to Table 2). The following three characteristics were excluded from the multivariate analyses, as they were not found to be associated with a PRS request in the univariate analyses: type of registration, pediatric indication or formulation, and public health impact. The characteristics “ATMP” and “exceptional medicinal products,” while associated with a PRS request at the significance level set for univariate analyses, were also excluded from multivariate analyses as their occurrence was associated with less than 10 dossiers in one group. As expected, missing values were not observed due to the exhaustivity of the database.

The results of the final logistic regression model are presented in Table 3. Overall, four characteristics were independently associated with a request for a PRS. First, medicinal products indicated in neurology (ORa = 2.34, 95 percent CI = [1.08–5.08]); pulmonology (ORa = 4.18, 95 percent CI = [1.87–9.35]); or endocrinology (ORa = 2.44, 95 percent CI = [1.07–5.56]) were more likely to receive a PRS request from HAS compared to dossiers that were not from the corresponding therapeutic area. Furthermore, compared to products with an important clinical benefit score, products that obtained a moderate or mild clinical benefit score were associated with an increased probability of PRS request (ORa = 2.90, 95 percent CI = [1.35–6.24], and ORa = 6.35, 95 percent CI = [3.02–13.40], respectively). Additionally, products with a major to moderate or minor clinical added value score were more likely to have a PRS request than products with no clinical added value score (ORa = 2.63, 95 percent CI = [1.24–5.60], and ORa = 2.15, 95 percent CI = [1.15–3.99], respectively). Finally, products that were previously available in France through an early access program were also associated with a higher probability of PRS request (ORa = 1.87, 95 percent CI = [1.03–3.37]), compared to those which were not.

**Table 3.** Characteristics associated with a post-registration study request: estimates from the final multivariate analysis

Characteristic	Adjusted odds ratio [CI_95%_]
**Indication type**	
*Extension of indication*	Reference
*First indication*	1.47 [0.87–2.49]
**Therapeutic area^a^**	
*Not Oncology*	Reference
*Oncology*	0.62 [0.33–1.20]
*Not Neurology*	Reference
*Neurology*	2.34 [1.08–5.08]
*Not Endocrinology*	Reference
*Endocrinology*	2.44 [1.07–5.56]
*Not Pulmonology*	Reference
*Pulmonology*	4.18 [1.87–9.35]
**Orphan designation**	
*No*	Reference
*Yes*	1.29 [0.71–2.35]
**Previous early access program**	
*No*	Reference
*Yes*	1.87 [1.03–3.37]
**Clinical benefit**	
*Important*	Reference
*Moderate*	2.90 [1.35–6.24]
*Mild*	6.35 [3.02–13.40]
**Clinical added value**	
*Absent*	Reference
*Minor*	2.15 [1.15–3.99]
*Major, important, or moderate*	2.63 [1.24–5.60]

Abbreviations: 95%CI, Confidence interval at a 95% level.

a The variable therapeutic area has been coded in four dummy dichotomous variables. For a specific therapeutic area (e.g., oncology), the odds ratio must be interpreted relatively to all the other dossiers that are not from the corresponding area.

Indication type and orphan designation status were not found to be associated with a PRS request at a 5 percent nominal alpha level. However, they were retained in the final multivariate model, as they contribute sufficiently to the fit of the model, as per the variable selection criteria described above.

No first-order interaction term was included in the final multivariate logistic regression model as including such parameters did not contribute to improve sufficiently the fit of the model (see detail in the online supplementary file eText 1).

## Discussion

This analysis suggests that several characteristics may be associated with a PRS request. On the one hand, the probability of a PRS request seems to increase with the diminution of the clinical benefit (ORa = 2.90, 95 percent CI = [1.35–6.24, for “moderate” clinical benefit and ORa = 6.35, 95 percent CI = [3.02–13.40], for “mild” clinical benefit, compared to an “important” clinical benefit score). According to the HAS doctrine (3), a “moderate” or “mild” clinical benefit may suggest suboptimal robustness of the evidence and/or limited effect size. On the other hand, the analysis indicates that innovative products, with clinical potential but uncertainties, could also be associated with a higher probability of PRS request. Products with a clinical added value indeed had a higher probability to obtain a PRS request than products with no clinical added value as recognized by HAS (ORa = 2.63, 95 percent CI = [1.24–5.60], for “major” to “moderate” clinical added value and ORa = 2.15, 95 percent CI = [1.15–3.99], for “minor” clinical added value). Since products with a “mild” to “moderate” clinical benefit rarely obtain a positive clinical added value score (43), these two profiles are deemed distinct. Additionally, products accessible through an early access program, which is restricted to pharmaceuticals presumed to be innovative, were also associated with a higher probability of a PRS request (ORa = 1.87, 95 percent CI = [1.03–3.37]). A plausible interpretation of the results is consistent with our working hypothesis. The results suggest two distinct profiles of PRS requests: i. products for which the benefit seems questionable and ii. innovative products for which a substantial benefit is expected but for which uncertainties remain. However, PRS requests seem to be more closely associated with the clinical benefit score than with the clinical added value score. This finding suggests that PRS requests are primarily prompted by uncertainties regarding benefit(s) and evidence.

On another note, this analysis reveals that the probability of a PRS request may depend on the therapeutic area. Products indicated in neurology, pulmonology, or endocrinology were indeed more likely to obtain a request for PRS by HAS compared to products licensed in other areas (ORa =2.34, 95 percent CI = [1.08–5.08], ORa = 4.18, 95 percent CI = [1.87–9.35] and ORa = 2.44, 95 percent CI = [1.07–5.56], respectively). Although these findings were unexpected, one possible explanation is that in these medical areas, there are often multiple comparators but rarely active-controlled RCTs. The absence of head-to-head comparison may contribute to uncertainties surrounding the optimal treatment pathway(s), thereby justifying a request for studies based on real-world data. For instance, in relapsing multiple sclerosis, several immunomodulators are recommended, but most of the randomized trials used interferon or placebo as the study comparator (44). To obtain more insights into optimal treatment sequences, in the last decade, HAS has almost systematically requested PRS for medicinal products licensed in this indication (45–50).

While oncology medicinal products are frequently launched with limited evidence and/or through a conditional marketing approval (50), no increased probability of PRS request was observed compared to products indicated in other areas (ORa = 0.62, 95 percent CI = [0.33–1.20]). This finding might be partially explained by the dynamic nature of treatment pathways in oncology. The rapid evolution of these pathways means that once data becomes available, it may quickly become outdated. HAS might therefore hesitate to request a PRS knowing that the care pathway is likely to change quickly due to the product soon obtaining an extension of indication in an earlier line of treatment. As PRS are requested with the intent to close an evidence gap and because oncology products are often launched with limited evidence (27), this finding might also suggest that uncertainties in oncology are considered more acceptable by HAS than in other therapeutic areas due to high medical need.

The primary strength of this study lies in its reliance on the analysis of a standardized, reliable, and exhaustive database. Consequently, for the study period (2016–2021), the estimated statistics can be considered nearly unbiased, offering values close to the true values of the population of relevant dossiers. Moreover, as the study spans 7 years of recent HAS activity, estimations derived from this sample of dossiers can be considered highly predictable for the foreseeable future. However, the main limitation of the study stems from its retrospective analysis of an administrative database that captures pre-specified characteristics, some of which may not be the most relevant for the purpose of the study. As outlined in Table 1, characteristics such as the clinical benefit and the clinical added value are graded by weighting numerous determinants. Consequently, analysis of these characteristics is somewhat constrained by the fact they are aggregates of multiple determinants, making it challenging to differentiate the individual components. Another limitation is related to the combination of most of the therapeutic areas into one category (i.e., “other therapeutic areas”). This combination was performed as it was not reasonable to estimate 31 regression coefficients (with corresponding standard errors) in a multivariate logistic regression model for only one characteristic. Each of these combined therapeutic areas represent less than 5 percent of the dossiers during the study period. Therefore, the probability of missing a significant effect estimated with a high level of precision was probably low. Nonetheless, we cannot exclude the possibility that an association(s) between PRS request and other therapeutic areas could exist.

This study represents the first statistical analysis aimed at identifying the characteristics associated with requests for additional studies based on real-world data during the appraisals of medicinal products by HAS. These findings will assist health technology developers to better anticipate data generation to quickly address uncertainties identified by HAS. Additionally, it will also support HAS in continuing to adapt its request strategy according to the medicinal product’s characteristics. The design of the study and the outcomes requested by HAS may indeed vary depending on the product’s characteristics. To provide a more in-depth understanding of the two profiles and the role of PRS in the French HTA system, a supplementary study assessing the outcomes of these PRS requests and the impact of these results on the reassessments conducted by HAS would be relevant. Finally, it is noteworthy that while continuous monitoring of real-world data, as planned by HAS for CAR-T cells, can be seen as a relevant model for assessing innovation, it is also a time- and resource-intensive endeavor that requires the availability of a high-quality registry.

Although this analysis was carried out using French data, the results and conclusions on post-licensing data generation may have relevance at the international level. First, post-licensing data are frequently generated at the international level, and national or regional studies are less common because of their feasibility. In addition, despite the structural differences between national reimbursement systems, HTA bodies share many principles when assessing the level of acceptable uncertainty of clinical data generated by healthcare technology manufacturers, as these principles are systematically grounded in the evidence-based medicine framework. This is illustrated by the strength of HTA networks at the international level, and especially with the introduction of the EU 2021/2282 regulation, which will soon lead to the conduct of joint clinical assessments and joint scientific consultations at the European level. Therefore, the key findings to plan RWE identified in this study are probably also relevant for other HTA frameworks, including for outcomes-based managed entry agreements like those implemented in Italy (51 *;* 52) or England (19 *;* 20 *;* 53).

Overall, this article further reinforces the need for international collaborations on PLEG, including between regulators and HTA bodies, which could enhance the efficiency of data planning and avoid duplication of national effort. This is particularly important for rare diseases where national registries may face limitations due to the prevalence and incidence of these diseases. The Joint Scientific Consultation introduced by the EU Regulation 2021/2282 on HTA that will start in 2025 should provide a further opportunity for multi-stakeholder discussion on overall data generation plans, including postlaunch.

## Supporting information

Fernandez et al. supplementary material Fernandez et al. supplementary material

## Supplementary material

The supplementary material for this article can be found at http://doi.org/10.1017/S0266462324000291.
