# Supplementary material for: Can requests for real-world evidence by the French HTA body be planned? An exhaustive retrospective case–control study of medicinal products appraisals from 2016 to 2021
Source: Int J Technol Assess Health Care. 2024 May 17;40(1):e33. doi: 10.1017/S0266462324000291 (PMC11569913; doi:10.1017/S0266462324000291)
Supplement: Fernandez et al. supplementary material [file S0266462324000291sup001.docx]

**Fernandez et al. Can requests for real-world evidence by the French HTA body be planned? An exhaustive retrospective case-control study of medicinal products appraisals from 2016 to 2021. Online Supplementary file**

**Supplementary eTable 1. List of all characteristics available in EVAMED**

| **Data available in EVAMED for clinical HTA dossiers** | **Extracted to set up the database** | **Included in the univariate analysis** | **Included in the multivariate analysis** |
| --- | --- | --- | --- |
| **General information** | | | |
| Dossier number | X |  |  |
| Indication type | X | X | X |
| Registration type | X | X | X |
| International Nonproprietary Name (INN) | X |  |  |
| Name of the health technology developer | X |  |  |
| Address of the health technology developer |  |  |  |
| Contact details of the health technology |  |  |  |
| Health economic dossier associated |  |  |  |
| **Timelines** | | | |
| Date of the marketing authorisation |  |  |  |
| Date of submission |  |  |  |
| Date of pre-submission |  |  |  |
| Date of validation |  |  |  |
| Date of examination |  |  |  |
| Date of first adoption |  |  |  |
| Start date of the contradictory phase |  |  |  |
| Date of oral hearing or examination of written observation |  |  |  |
| Date of the final appraisal | X |  |  |
| Date of publication | X |  |  |
| Overall assessment and appraisal time | X |  |  |
| **Description of the dossier** | | | |
| Therapeutic indication | X |  |  |
| Therapeutic Area | X | X | X |
| ATC code | X |  |  |
| Individual national code of the medicinal product (CIP) |  |  |  |
| Pediatric indication | X | X |  |
| Conditional marketing authorisation / marketing authorisation under exceptional circumstances | X | X |  |
| Previous national early access program | X | X | X |
| Orphan designation | X | X | X |
| Advanced Therapy Medicinal Products (ATMP) | X | X |  |
| Exceptional medicinal therapy | X | X |  |
| Generic, biosimilar or essentially similar medicinal product | X |  |  |
| Companion diagnostic test | X |  |  |
| Associated medical act | X |  |  |
| Rare disease | X |  |  |
| Previous early dialogue |  |  |  |
| Presumed innovation | X | X |  |
| **Appraisals** | | | |
| Clinical Benefit score | X | X | X |
| Public Health impact criteria | X | X |  |
| Clinical added value score | X | X | X |
| Post-registration study | X |  |  |
| Post-registration study number | X |  |  |
| Request of oral hearing |  |  |  |
| Impact of the contradictory phase |  |  |  |

**Supplementary eText 1. Search for effect modification (first order interaction terms). Methods and results.**

1. **Satistical analysis**

Starting from the final multivariate model selected (i.e., see Table 3 in the paper), adding one by one all possible first order interaction terms was tested to search for possible candidates for effect modification to be included into the multivariate logistic regression model, at a nominal alpha level of 5%. Characteristics for which this test for interaction was significant were included into a first multivariate logistic regression model to check if the level of significance of the interaction terms between all the combination of the modalities of the considered characteristics was still met at an alpha level of 5% after adjustment on the other primary effects and other interaction terms. As only one interaction term between one combination of modalities of two variables (among 8 possibles combination of modalities) was found to be at a p-value below 0.05, a global likelihood ratio-test was performed between the model without any interaction term and the model with an interaction between the two variables as a whole to check if adding globally an interaction between the two variables (which requires 8 supplementary regression coefficients to fit) led to a sufficient improvement in model fit.

1. **Results**
2. Tests of all possible interaction terms one by one

| **Interaction term** | **p-value** |
| --- | --- |
| Clinical added value x Orphan Designation | 0.03* |
| Indication type x Orphan Designation | 0.04* |
| Therapeutic area x Clinical added value | 0.049* |
| Clinical added value x Previous early access program | 0.055 |
| Therapeutic area x Orphan Designation | 0.06 |
| Previous early access program x Orphan designation | 0.10 |
| Indication type x Clinical benefit | 0.10 |
| Clinical benefit x Orphan Designation | 0.10 |
| Therapeutic area x Previous early access program | 0.37 |
| Indication type x Previous early access program | 0.40 |
| Clinical benefit x Previous early access program | 0.42 |
| Clinical added value x Clinical benefit | 0.46 |
| Indication type x Therapeutic area | 0.61 |
| Indication type x Clinical added value | 0.85 |
| Therapeutic area x Clinical benefit | 0.98 |

**Note:** Interaction tests results were ranked in increasing order of p-value to improve readability

From these one-by-one analyses, three interaction terms can be retained for multivariate regression analyses: clinical added value x orphan designation, indication type x orphan designation and therapeutic area x clinical added value.

1. Significance of interaction terms between all the combination of the modalities of the retained candidates when incorporated into the multivariate logistic regression model

| **Interaction term** | **p-value** |
| --- | --- |
| Clinical added value (Major, important or moderate) x Orphan Designation (Yes) | 0.95 |
| Clinical added value (Minor) x Orphan Designation (Yes) | 0.08 |
| Indication Type (Extension of indication) x Orphan Designation (Yes) | 0.06 |
| Therapeutic area (Oncology) x Clinical Added Value (Minor) | 0.21 |
| Therapeutic area (Neurology) x Clinical Added Value (Minor) | 0.77 |
| Therapeutic area (Pulmonology) x Clinical Added Value (Minor) | 0.27 |
| Therapeutic area (Endocrinology) x Clinical Added Value (Minor) | 0.36 |
| Therapeutic area (Oncology) x Clinical Added Value (Major, important or moderate) | 0.04* |
| Therapeutic area (Neurology) x Clinical Added Value (Major, important or moderate) | 0.58 |
| Therapeutic area (Pulmonology) x Clinical Added Value (Major, important or moderate) | 0.37 |
| Therapeutic area (Endocrinology) x Clinical Added Value (Major, important or moderate) | 0.96 |

After incorporating potential candidate for first order interactions into the multivariate logistic regression model, the only potential significant interaction at a nominal alpha level of 0.05 is between therapeutic area and clinical added value, only for one of the 8 possible combinations of the modalities of the two variables.

1. Global likelihood-ratio test between the multivariate regression model without any interaction terms and a model with an interaction between Therapeutic area and Clinical added value as a whole

After multivariate analyses, only one interaction term between only one combination of the modalities of two variables (among 8 possibles combination) was found to be at a p-value below 0.05. Therefore, a global likelihood-ratio test to check if including an interaction term between therapeutic area and clinical added value as a whole leads to a significant improvement of model fit was performed.

The p-value of the likelihood-ratio test is 0.054 (above the nominal alpha level of 0.05), which means that incorporating the interaction between therapeutic area and clinical added value (8 more regression coefficients to fit compared to the model without interaction) within the model does not lead to a sufficient improvement of fit compared to not include the interaction.
